# Doping effect in graphene-graphene oxide interlayer

**DOI:** 10.1038/s41598-020-65263-y

**Published:** 2020-05-19

**Authors:** Mohd Musaib Haidari, Hakseong Kim, Jin Hong Kim, Minwoo Park, Hoonkyung Lee, Jin Sik Choi

**Affiliations:** 1 Department of Physics, Konkuk University, Seoul, 05029 Korea; 2 Korea Research Institute of Standards and Science (KRISS), Daejeon, 34113 Korea

**Keywords:** Materials science, Nanoscience and technology

## Abstract

Interlayer coupling in graphene-based van der Waals (vdW) heterostructures plays a key role in determining and modulating their physical properties. Hence, its influence on the optical and electronic properties cannot be overlooked in order to promote various next-generation applications in electronic and opto-electronic devices based on the low-dimensional materials. Herein, the optical and electrical properties of the vertically stacked large area heterostructure of the monolayer graphene transferred onto a monolayer graphene oxide film are investigated. An effective and stable p-doping property of this structure is shown by comparison to that of the graphene device fabricated on a silicon oxide substrate. Through Raman spectroscopy and density functional theory calculations of the charge transport characteristics, it is found that graphene is affected by sustainable p-doping effects induced from underneath graphene oxide even though they have weak interlayer interactions. This finding can facilitate the development of various fascinating graphene-based heterostructures and extend their practical applications in integrated devices with advanced functionalities.

## Introduction

Various two-dimensional (2D) van der Waals (2D-vdW) materials have been investigated for promising applications in futuristic electronic systems^1–4^. Owing to the experimental and theoretical studies on 2D-vdW materials, unprecedented developments in fundamental science as well as the industrial fields have been accomplished. As 2D-vdW material-based components such as transistors, memories, energy harvesters, and sensors have shown immense potential for applications in advanced logic systems, extensive research efforts have been focused on overcoming the performance limits of the single 2D material^5–8^. Among the different types of 2D-vdW materials investigated to realize optimal devices, vertically combined structures are promising candidates to achieve high-efficiency logic components and overcome current performance limitations of the individual 2D-vdW materials^4,9^.

Owing to the atomically flat and well-defined clean interfaces, vertical hetero-structures allow unlimited combinations that can be optimized for various conceptual devices based on the demands. As a result, many devices such as graphene barristors^10^, solar cells^11^, and light-emitting diodes^12^ have been introduced to improve and optimize the devices for a wide range of industrial applications. However, in the 2D-vdW heterostructures, interlayer coupling can usually cause unintended phenomena during the operation of the designed device. Therefore, understanding the physical and chemical interactions between the vertically combined vdW heterostructures is key to the design and modulation of device operation based on the requirements. For investigating the interlayer coupling effect in combined structures, graphene and graphene oxide (GO) have an advantage, as the intrinsic properties of both materials can be modulated by an external stimulus and/or chemical adsorption. Therefore, the interlayer interaction between graphene and GO films in a heterostructure can be clearly identified by comparing the experimental results with theoretical expectations.

In this study, the interlayer coupling effect on the electrical charge transport and Raman spectroscopy measurements is investigated in vertically stacked graphene on GO films. The similarities and differences from the individual materials are carefully identified through experimental and computational studies. Using chemical vapor deposition (CVD) grown monolayer graphene and GO prepared via photochemical treatment, graphene/GO heterostructure was obtained by transferring graphene onto GO^13–15^. Three types of field-effect transistor (FET) devices were fabricated in each area of the graphene, GO and graphene/GO sheets to compare the electrical and optical properties. The experimental strategies employed in this study can be utilized not only to reveal the physical origins of graphene doping effects in vdW hetero structures but also for designing new 2D vdW structures for applications in various conceptual electronic systems as well as their optimization for use in electrical and optical components.

## Results

**Figure 1:**
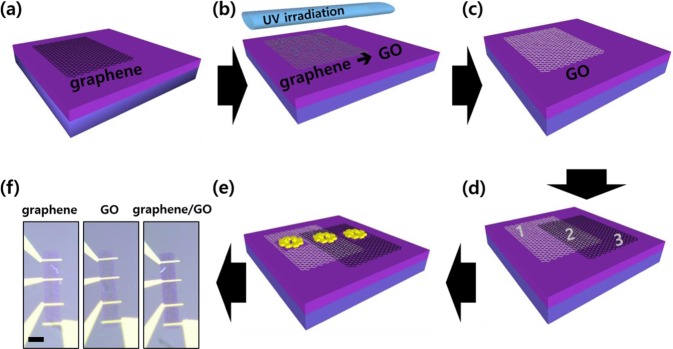
Fabrication of graphene-, GO-, and graphene/GO-based FET devices. (**a**) CVD-fabricated monolayer graphene transferred on thermally oxidized SiO_2_/Si substrate. The thickness of SiO_2_ is 300 nm. (**b**) UV irradiation to oxidize the monolayer graphene. (**c**) Monolayer GO film on SiO_2_/Si substrate. (**d**) Monolayer graphene transferred on the partial areas of GO sheet and SiO_2_ substrate. Regions 1, 2, and 3 represent GO, graphene/GO, and graphene areas, respectively. (**e**) Three types of FET devices fabricated on each region in the SiO_2_ substrate. (**f**) Optical microscopy image of the FET devices on the three regions. The scale bar is 10 μm.

Figure 1 shows a schematic of the fabrication of graphene, GO, and graphene/GO on the SiO_2_/Si substrate. As shown, GO films are prepared by UV irradiation of the graphene film deposited on the SiO_2_/Si substrate. Excimer UV irradiation with a wavelength of 172 nm is used for oxidation at room temperature at ambient conditions within 1 min without wet chemical treatment (Fig. 1b,c). Thereafter, the second monolayer graphene is transferred on the SiO_2_/Si substrate to partially cover both GO and SiO_2_ surfaces. As a result, graphene, GO, and graphene/GO regions were prepared on the SiO_2_/Si substrate (Fig. 1d). The thicknesses of these regions were analyzed using the line profile from AFM topography (Supplementary information, Fig. S1). Three types of FET devices are fabricated using the standard cleanroom procedure as shown in Fig. 1e. All devices are fabricated with an identical pattern using electron beam lithography, and undesired areas are etched using a dry etching system with O_2_ plasma. Cr (5 nm) and Au (90 nm) are sequentially deposited to provide an electrical contact on FET devices. The three types of FET devices with transport channels denoted as graphene, GO, and graphene/GO are prepared as shown in Fig. 1f.

Notably, of the various available methods to obtain GO films^16,17^, the most conventional method does not employ UV-irradiation, but instead involves dispersion using filtered graphite oxide flakes^18^. In these methods, GO is obtained via several steps of chemical treatment. The process not only requires several hours for completion, but the treated surface is also covered with inhomogeneous functional groups such as hydroxyl (-OH), carboxyl (-COOH), and epoxide (-O-) groups. Because the electrical properties of CVD graphene can be easily modified by charged impurities or chemical components dispersed on the GO surface as well as the substrate, UV-irradiation technique is employed in this study to obtain a clean GO surface (Supplementary information, Fig. S2), which is comparable to that obtained by other methods, as we reported in our previous work^19^. Moreover, the prepared GO sheets may only contain the epoxy groups as reported by Mulyana *et al*.^20^.

**Figure 2:**
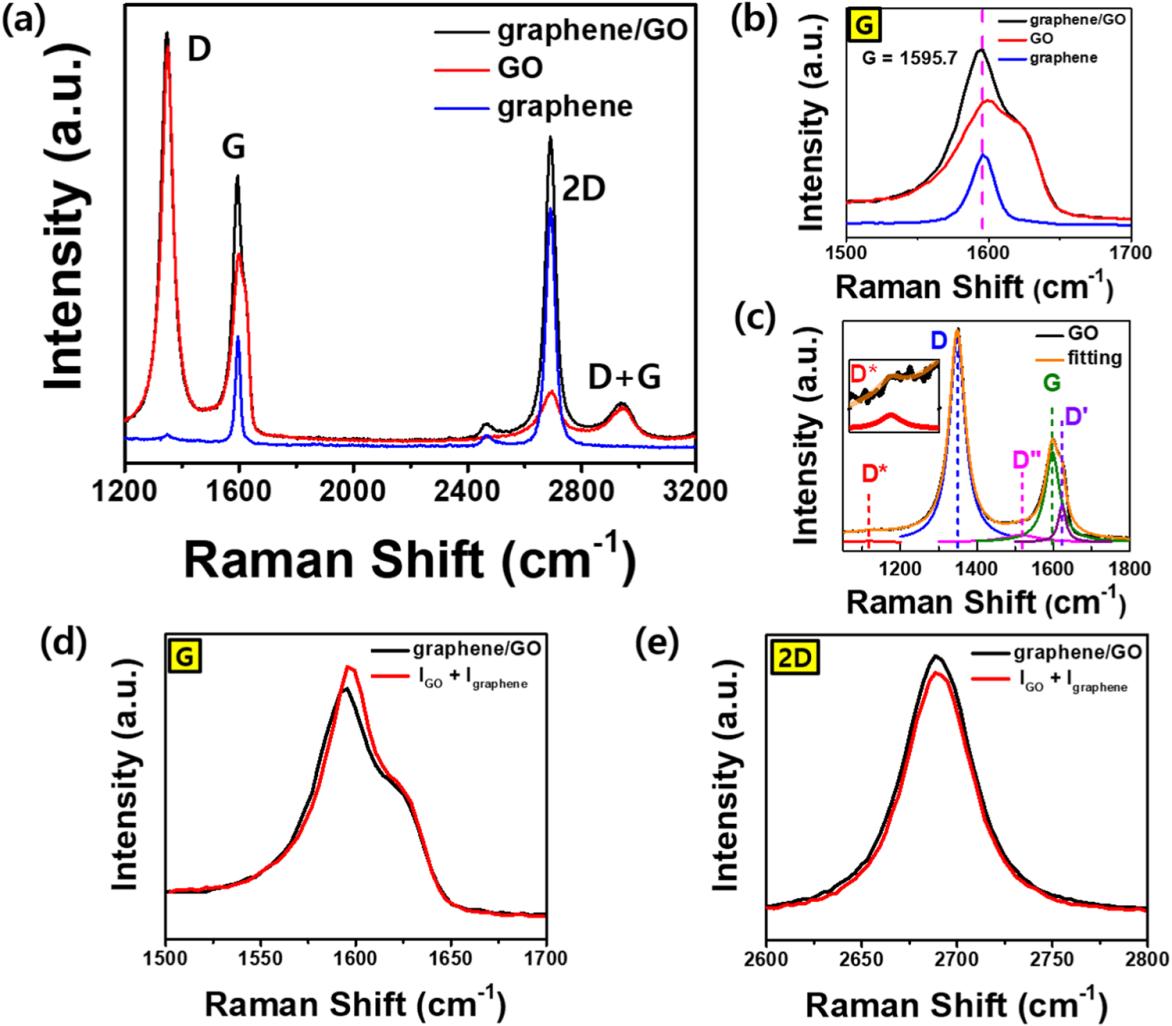
Raman spectra of graphene, GO, and graphene/GO on SiO_2_ substrate. (**a**) Full spectra showing four distinct peaks including D, G, 2D, and D + G peaks in the Raman spectra of graphene (blue curve), GO (red curve) and graphene/GO (black curve). (**b**) G-peak spectra of graphene, GO, and graphene/GO. Peak at 1595.7 cm^−1^ is designated using a magenta colored dashed line. (**c**) Gaussian fitting results for the Raman spectra of D and G peaks in GO (orange line) constituting the D (~1351 cm^−1^), D′ (~1623 cm^−1^), D″ (~1515 cm^−1^), G (~1597 cm^−1^), and D^*^ (~1120 cm^−1^) peaks. (**d**) Comparison between the G-peak spectra obtained experimentally from graphene/GO (black line) and a mathematical summation of the G-peak spectra of graphene and GO (red line). (**e**) 2D peaks observed on graphene/GO (black line) and a mathematical summation of the 2D-peak spectra of graphene and GO. In all Raman spectra, the incident laser with an excitation wavelength of 532 nm is used.

As shown in Fig. 2a, the distinct Raman spectra for the three defined regions (graphene, GO, and graphene/GO) are clearly identified. For a CVD-fabricated monolayer graphene, the intensity ratio of 2D/G in the Raman spectra is determined to be approximately 3, which indicates that the graphene is crystalized. However, in the UV irradiated region, the Raman characteristics change to those of the typical GO, as a high-intensity D peak (~1349 cm^−1^), broad G peak (~1595 cm^−1^), and discernible D + G peak (~2944 cm^−1^) are observed^21^. As shown in Fig. 2b, a slight blue shift in the G peak (1599.2 cm^−1^) of the GO sample is observed in comparison to that (1595.5 cm^−1^) of the graphene sample. Despite the changes in the Raman spectrum of graphene due to UV irradiation, the optical contrast of the graphene layer on SiO_2_ substrate did not change significantly, and the graphene was indistinguishable from GO under optical microscopy (Supplementary information, Fig. S3a). However, the surface energy of graphene increased due to the UV-oxidation process, which transforms the hydrophobic graphene to hydrophilic GO (Supplementary information, Fig. S3b).

For a detailed analysis of this behavior, the Raman spectrum of GO was fitted with multiple peak functions in the 1500–1700 cm^−1^ range. Fig. 2c shows the extracted G, D′, and D″ peaks from the GO spectrum with Gaussian function fitting^22^ (D′ and D″ bands are designated as D_2_ and D_3_, respectively, in others’ studies^23^). The calculated peak positions of G, D′, and D″ peaks obtained by fitting are 1597.1, 1623.3, and 1513.5 cm^−1^, respectively. These results are intriguing, and several possible explanations have been suggested for the blue shift of G peak in GO. One explanation is based on the double bonds and the other on the D′ band. In theoretical calculations, the alternating pattern of the single-double carbon bonds within the sp^2^ carbon ribbons yields blue-shifted high-intensity Raman bands^24^. Other studies have suggested that the merging of D′ band (~1620 cm^−1^) can also be partially responsible for this blue shift. The presence of D′ band can be explained by the disorder-induced phonon mode above the G band due to crystal defects^25,26^.

Next, the Raman spectroscopy results for the graphene/GO region were examined. Interestingly, for graphene/GO, the Raman spectrum appears to be a simple mathematical summation of those for graphene and GO. To confirm this observation, the Raman spectrum of the graphene/GO region was analyzed by comparing it to the mathematical summation of the spectra obtained from graphene and GO regions. As shown in Fig. 2d,e, the experimental results for G and 2D peaks coincide with their summation data. This implies that the layers of graphene and GO maintain their characteristics (based on Raman spectra) upon forming a junction. Moreover, the additivity of the Raman spectrum of graphene/GO may be due to non-contact with molecular distances between graphene and GO layers. Through the AFM topography analysis, we have confirmed that the interlayer distance is less than 1 nm (Supplementary information, Fig. S1). Notably, the Raman spectra of graphene/GO such as peak positions and intensities are very uniform and homogeneous in the entire area (Supplementary information, Fig. S4). This unique characteristic of graphene/GO is interesting because the Raman spectrum of the layer-by-layer-transferred and twisted bilayer graphene generally shows a largely distinct feature according to the crystal orientations of the two graphene layers due to interlayer coupling effects^21,27^. However, the typical Raman spectrum for the twisted bilayer graphene is not observed in the graphene/GO structure in the present study^28–31^. Thus, it is concluded that the observation of the summation of individual Raman spectra of the two stacked layers is a unique property of the vdW heterostructures of G and GO, without any interlayer interactions^32^.

**Figure 3:**
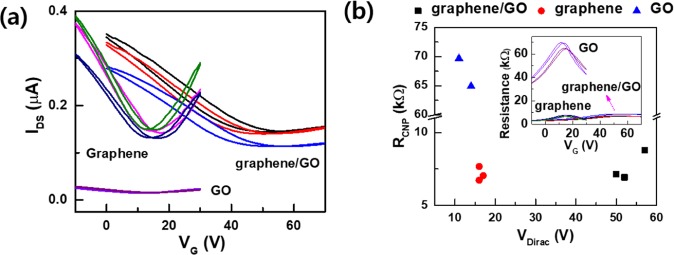
Electrical transport properties of the three types of FET devices. (**a**) Back-gate-induced FET characteristics of graphene, GO, and graphene/GO. (**b**) Resistances at the charge neutral point (CNP) as a function of Dirac-point voltage. Inset of (**b**) shows the channel resistance plot as a function of back gate voltage.

Figure 3 exhibits the electrical transport properties of graphene, GO, and graphene/GO. For the analysis of the electrical transport properties, the current level, carrier mobility, and resistance value were evaluated at the charge neutral point (CNP) through the back gate voltage sweep between −10 V to 30 V for graphene and GO, and from 0 V to 70 V for graphene/GO (Fig. 3(a)). Detailed parameters including the Dirac voltage (*V*_*Dirac*_), carrier density (*n*), mobility (*µ*), and resistance in CNP (*R*_*CNP*_) for each device are listed in Table 1. Interestingly, graphene and the monolayer GO have identical values for *V*_*Dirac*_, but the current level is significantly different by an order of magnitude. Both graphene and graphene/GO exhibit similar current levels, but wider transfer curve characteristics and higher Dirac point are observed for the graphene/GO device. To confirm the repeatability and reliability of these trends, additional FET devices were fabricated and characterized by back-gate-induced measurements. All devices showed the same tendencies with respect to variations in V_Dirac_, current level, and I–V curve slope (Supplementary information, Fig. S5).

**Table 1:** Electrical properties measured from the FETs fabricated on graphene, GO, and graphene/GO areas.

	Graphene/GO	Graphene	GO
V_Dirac_ (V)	53	16	12
R_CNP_ (kΩ)	7.6	7.1	67.3
n (cm^−2^)	3.43E + 12	1.11E + 12	2.57E + 12
µ (cm^2^V^−1^S^−1^)	530	1328	90

*V*_*Dirac*_ is the Dirac voltage, *R*_*CNP*_ is the resistance at CNP, *n* is the carrier density, and *µ* is the mobility.

Considering the electrical transport results, the resistance of graphene increases ten times, while *V*_*Dirac*_ is almost maintained at the original position after UV irradiation. When graphene is transferred onto GO, the graphene channel retains an almost similar current level as that in the case when it is transferred onto the SiO_2_ substrate, but the Dirac point shifts from 14 V for graphene/SiO_2_ to 54 V for graphene/GO, which indicates that graphene on GO is highly hole-doped by the GO layer. Qualitatively, this is inconsistent with the negligible blue-shift of the G peak in Raman analysis (Fig. 2(b)), but the doping effect appears to be large. It is speculated that the high p-doping effect is due to the high charge accumulation between graphene and GO layers, but no discernible features for the chemical bonding between graphene and GO are observed. The positive Dirac voltage for graphene/SiO_2_ indicates that there is an electrostatic potential difference between the two independent layers without chemical bonding. The carrier densities of GO, graphene, and graphene/GO were obtained from the following equation by fitting.

The resistance of the transport channel is defined as Eq. (1)^33^

1 $${R}_{ch}=\frac{L/W}{q\mu \sqrt{{\left\{\frac{{C}_{ox}({V}_{G}-{V}_{Dirac})}{q}\right\}}^{2}+{n}^{2}}}$$

where *R*_*ch*_ is the channel resistance of graphene, *L* and *W* are the length and width of the graphene channel, *q* is the Coulomb unit charge, *C*_ox_ = *ε*_ox_*ε*_0_/*t*_ox_ is the gate oxide capacitance per unit area, *ε*_ox_ = 3.9 is the dielectric constant of SiO_2_, *ε*_0_ is the vacuum permittivity, and *t*_ox_ is the thickness of the oxide film (300 nm). *V*_Dirac_ is the Dirac point estimated at the conductance minimum of the graphene channel during the gate voltage (*V*_G_) sweep, *n* is the carrier density, and *μ* represents the mobility of graphene FET. A higher carrier density of GO in comparison to that of graphene is due to the functionalities on the surface of GO. Carrier density in graphene/GO is almost equal to the summation of the carrier densities in graphene and GO regions.

Figure 3(b) shows the variation in *R*_*CNP*_ as a function of *V*_*Dirac*_ distributions for each channel. Although the CNP position increases more than three times for graphene/GO in comparison to that for graphene and the resistance of GO is higher than that of graphene, no significant change is observed in the resistance of graphene/GO compared to graphene. Furthermore, the electrical properties of GO and graphene/GO exhibit excellent stabilities without any noticeable changes in the mobility and resistance for a year even when these are kept at ambient conditions.

**Figure 4:**
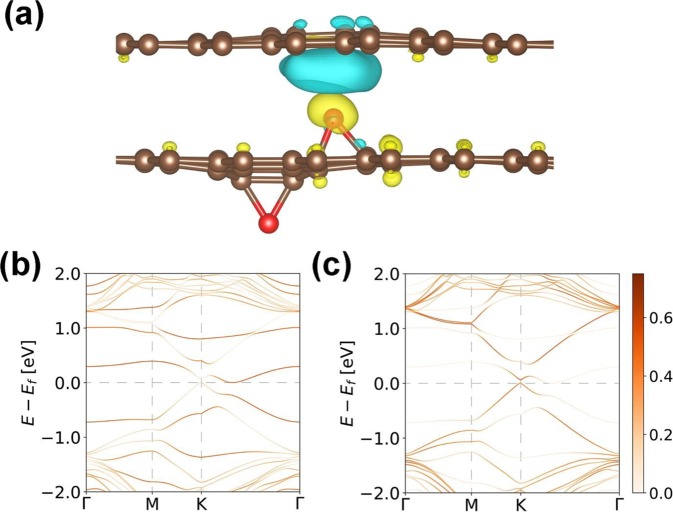
DFT calculations. (**a**) Difference in the charge densities of graphene and GO obtained from DFT calculations when the iso-surfaces of the charge densities are contoured at a level of ±0.0005 eÅ^−3^. Brown and red dots indicate the carbon and oxygen atoms, respectively, and yellow and blue colors indicate electron accumulation and depletion, respectively. Calculated fat band of (**b**) GO and (**c**) graphene on GO. Fermi level is set to zero.

To explain the high doping effect of G/GO, first-principles calculations were performed for the structure of graphene/GO using density functional theory (DFT)^34^ implemented in the Vienna Ab-initio Simulation Package (VASP) with a projector augmented wave (PAW)^35^ method, within the generalized gradient approximation (GGA) by Perdew, Burke, and Ernzerhof (PBE)^36^ theoretical calculations. Geometry optimization of the structures is carried out until the Hellmann–Feynman force acting on each atom is less than 0.01 eV/Å. We use 5 × 5 graphene and 5 × 5 graphene with 2 oxygens in the unit cell. The first Brillouin zone integration is performed using the Monkhorst–Pack scheme, and 5 × 5 × 1 k-point sampling for graphene on graphene oxide is used. Figure 4a shows the atomic structures with an overlay of the charge density difference at the graphene and GO iso-surfaces. The fat band analysis of graphene in the structure of GO (Fig. 4b) and graphene on GO (Fig. 4c) was performed. For the theoretical investigation of graphene on GO, the distance between graphene and GO layers is calculated as 3.2 Å (Fig. 4a), which indicates that there is no chemical bond between these layers. This is consistent with the Raman results. Nevertheless, there is a charge density difference between the two layers, which are depicted as electron depletion (blue) and electron accumulation (yellow) zones. This implies that an electron transfer occurs from the graphene sheet to GO through the oxygen groups, which causes a p-doping effect in the graphene layer^37,38^. Additionally, a Dirac cone-like feature is observed at the K-point with a very small gap (~0.05 eV) as shown in Fig. 4(c), which may be caused by the interaction between the O atoms and graphene.

## Conclusion

In conclusion, the graphene/GO structure is the thinnest vdW heterostructure known, which enables effective p-doping without chemical bonding. Raman spectroscopy measurements and electrical transport analysis, together with the corresponding simulations of the graphene/GO structure, confirm that there is no chemical bonding between graphene and GO layers. However, the electrons can flow from the graphene sheet owing to the difference in charge density, which is due to the presence of oxygen groups in GO, exhibiting a p-doping effect in the graphene layer. Additionally, these effects are observed for a long time under ambient conditions, affording almost perpetual p-doping effect on the surface of graphene deposited on GO. We suggest that the monolayer GO can be used as the thinnest hole-dopant supplier sheet for graphene without affecting its resistance value. Furthermore, by substituting the oxygen groups of GO by n-type-inducing functionalities, a graphene p-n junction can be obtained in a facile manner. We believe that our work could be stepping-stone for realizing various conceptual devices based on partial doping distributions using the 2D heterostructures and further could be contribute to engineering and modulating the electronic and optoelectronic applications.

## Methods

### Graphene synthesis

Monolayer graphene was grown on a Cu foil (Cu 99.999%; Puratronic, Alfa Aesar) at 1000 °C via tube-type thermal CVD (T-CVD) with a flow of CH_4_ and H_2_ (1:2) gas mixture for 20 min. The graphene on the Cu film was spin-coated with poly methyl methacrylate (950 PMMA Series Resists in Chlorobenzene; Microchem Corp.). Cu foil was etched by floating on 0.1 M ammonium persulfate ((NH_4_)_2_S_2_O_8_) aqueous solution. After rinsing several times with deionized water, the graphene film was transferred onto the SiO_2_/Si substrate. Then, the PMMA layer was removed using acetone, followed by rinsing and drying with isopropyl alcohol (IPA) and N_2_, respectively.

### UV irradiation

To oxidize graphene, Excimer UV irradiation with a wavelength of 172 nm and an illumination power of 30 mW/cm^2^ was used for oxidation at room temperature at ambient conditions without any wet chemical treatment. Optimized UV irradiation time is determined by Raman analysis. (Supplementary information, Fig. S6)

### Raman spectroscopy measurement

Raman spectroscopy measurements were performed using a WITec alpha 300 instrument (WITec GmbH, Ulm) using a diode laser with a wavelength of 532 nm. The laser power was set to 1 mW to prevent any destructive effects on graphene and GO atomic layers. The laser source was vertically exposed onto the device surface through a ×100 objective lens and the position of the sample was controlled within micrometer accuracy by a piezo stage.

### Electrical transport measurements

The I-V characteristics of the devices were investigated by electrical transport measurement using a vacuum probe station connected to Keithley 4200-SCS parameter analyzer. All electrical measurements were performed under a high vacuum of below 10^−6^ Torr. Si and SiO_2_ in the SiO_2_/Si substrate act as the gate and gate insulator, respectively. The I-V curves were obtained by measuring the current flow in the channels of the FET devices while controlling the bias voltage and gate voltage sweep.

## Supplementary information

Supplementary information.
