# Supplementary material for: Doping effect in graphene-graphene oxide interlayer
Source: Sci Rep. 2020 May 19;10:8258. doi: 10.1038/s41598-020-65263-y (PMC7237491; doi:10.1038/s41598-020-65263-y)
Supplement: Supplementary file 1 — Supplementary information. [file 41598_2020_65263_MOESM1_ESM.docx]

**Doping effect in graphene-graphene oxide interlayer**

Mohd Musaib Haidari^1+^, Hakseong Kim^2+^, Jin Hong Kim^1^, Minwoo Park^1^, Hoonkyung Lee^1^

, Jin Sik Choi^1^*

*^1^Department of Physics, Konkuk University, Seoul 05029, Korea*

*^2^Korea Research Institute of Standards and Science (KRISS), Daejeon 34113, Korea*

*E-mail: [jinschoi@konkuk.ac.kr](mailto:jinschoi@konkuk.ac.kr)

^+^These authors contributed equally to this work.

***1. AFM topography on GO, G/GO***


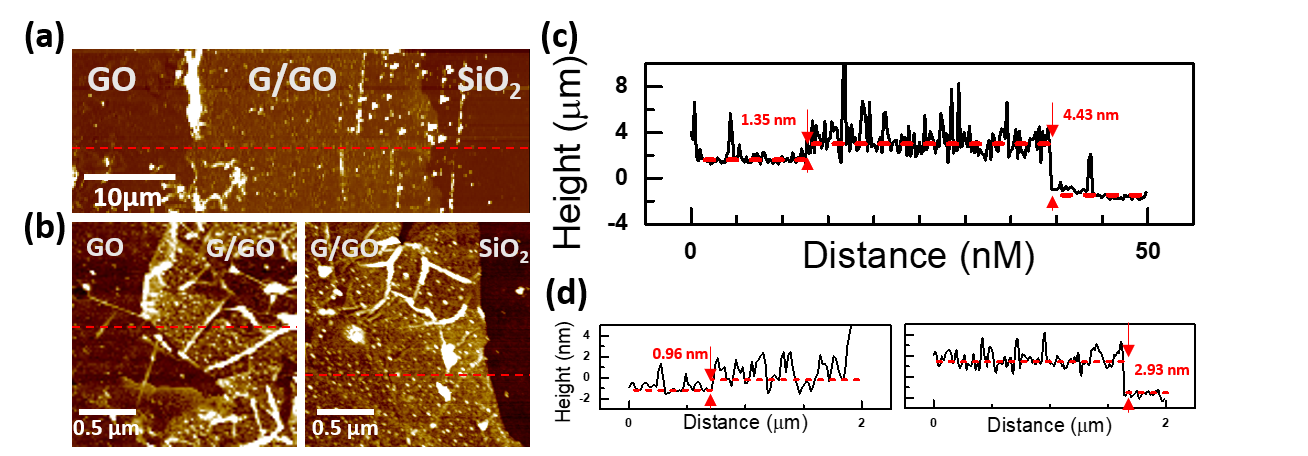


**Figure S1**. AFM topography results and height profiles on SiO_2_, graphene oxide (GO), and graphene on graphene oxide (G/GO) regions. (a) The topography of three regions (G, G/GO and SiO_2_) in one result. (b) The topography images from edges of GO and G/GO (left), and G/GO and SiO_2_ (right). (c) and (d) exhibit the height profile of red dashed lines in (a) and (b), respectively.

Figure S1 shows the AFM topography images of an area with three distinct regions: GO, G/GO, and SiO_2_. The height profiles from red dashed lines in (a) and (b) are shown in Figures S1(c) and (d), respectively. The height difference between GO and G/GO is 0.96 nm, as seen near the edge of the two regions; that between G/GO and SiO_2_ regions is 2.93 nm, as seen near their edge. Through these results, the thickness of G on the GO was calculated to be ~ 2 nm. Considering the height of the G measuring by using AFM (0.4 - 1.7 nm; Figure S1), the interlayer distance between G and GO seems to be less than 1 nm.

***2. AFM topography of GO***


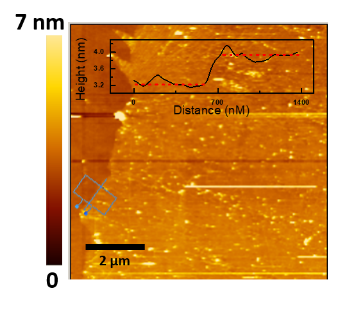


**Figure S2**. AFM topography image near the edge of the 45-s UV-irradiated GO. The inset shows the average line profile corresponding to the blue rectangle area. The average height of the graphene oxide was observed to be ~0.8 nm.

***3. Hydrophilicity of GO***

**
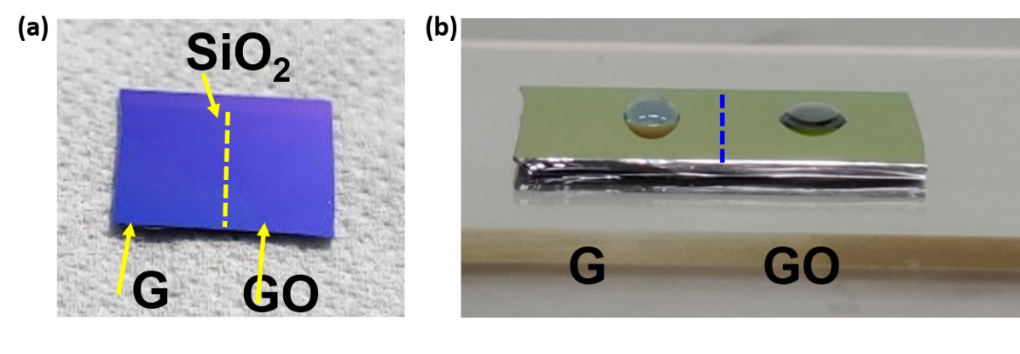
**

**Figure S3**. (a) Half-side oxidized graphene by using a metal mask. The red dotted line represents the border between graphene and graphene oxide, since it is indistinguishable by optical contrast. (b) Hydrophilicity of the graphene oxide by dropping DI water with comparison against the clearly hydrophobic graphene. The blue dotted line designates the border between graphene and graphene oxide.

***4. Raman analysis of on GO, G/GO***


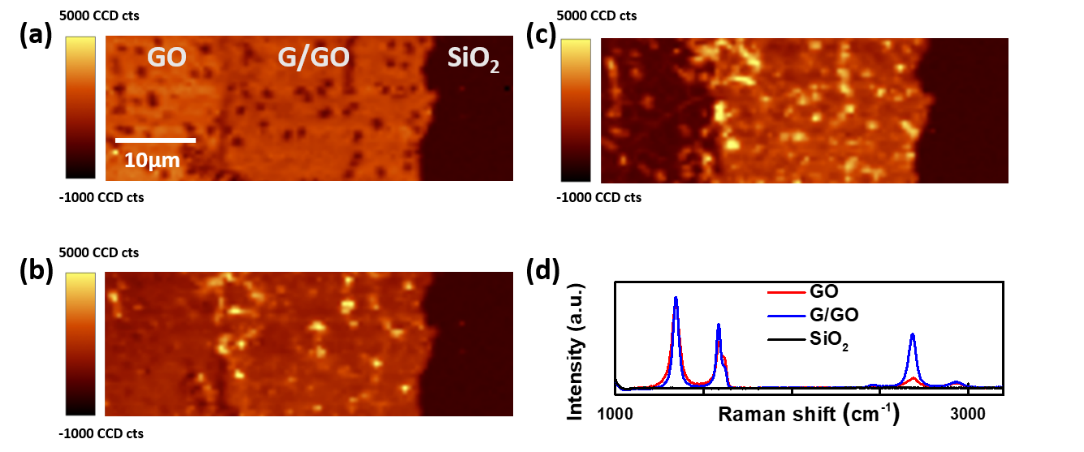


**Figure S4**. Raman data corresponding to the same area as in Figure S1. (a), (b), and (c) show the Raman mapping images for D-, G-, and 2D-peak, respectively. (d) exhibits the Raman spectra for SiO_2_ (black curve), GO (red curve), and G/GO (blue curve).

Figure S4 shows the Raman spectra and mapping images for SiO_2_, GO, and G/GO regions corresponding to the same area as in AFM topography images (Figure S1). Figures S4(a) and (b) show the D- and G-mapping images, respectively. The GO and G/GO regions have too minute contrast differences for obtaining D- and G-mapping images; however, in case of 2D-mapping (Fig S4. (c)), we can easily distinguish between GO, G/GO, and SiO_2_ regions, which supports the AFM data.

***5. Back-gate-induced characteristics of FET devices.***


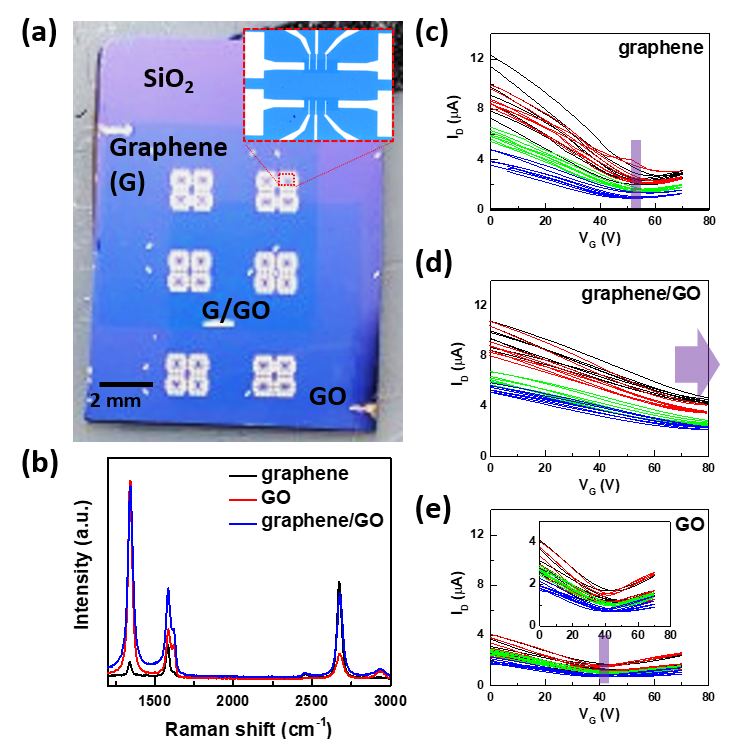


**Figure S5.** FET device fabrication and characterization. (a) Optical image of as-fabricated FET devices on graphene (G), G/GO, and GO. Inset shows a magnified image of the device channels. All devices are fabricated using the same pattern. (b) Raman spectra of the three areas of G, GO, and G/GO. (c-e) Back-gate-induced FET characteristics of graphene, G/GO, and GO, respectively. Different channel lengths are indicated by different colors. Transparent purple lines in Figs. S5c–S5e designate the position of V_Dirac_.

***6. Raman analysis of UV irradiated graphene***

**
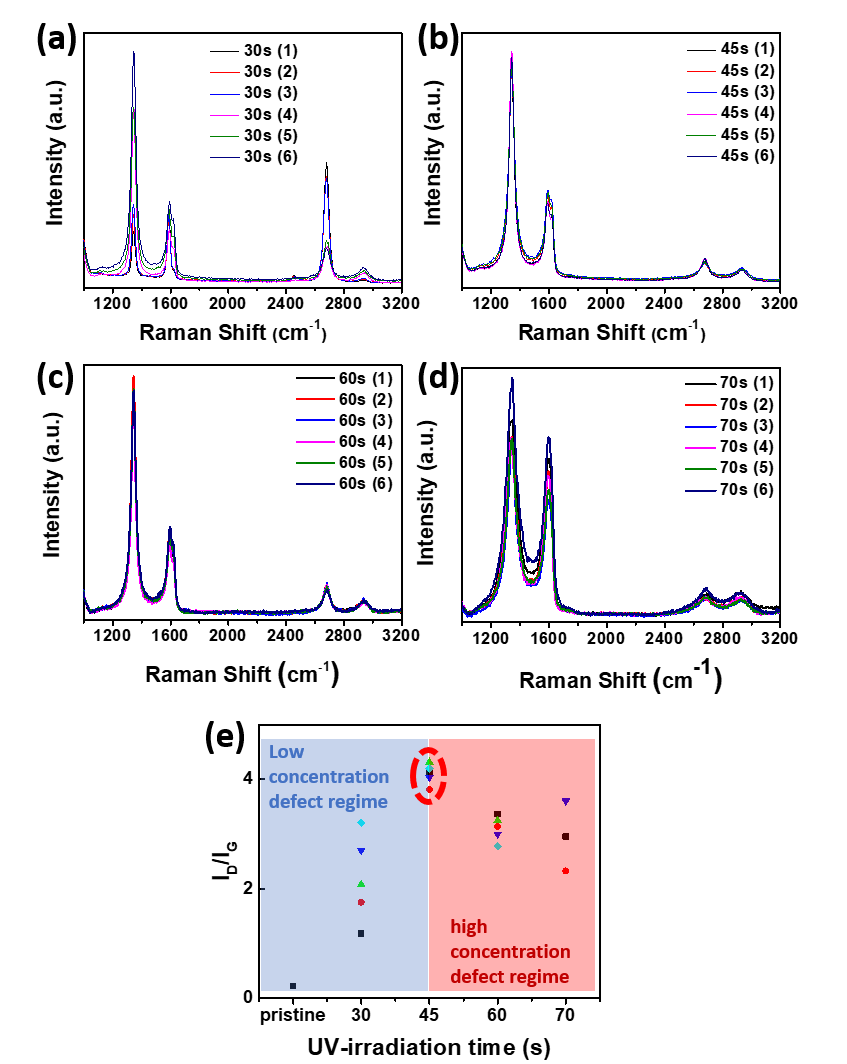
**

**Figure S6**. Raman analysis for UV-irradiation time optimization. (a-d) show Raman spectra from six arbitrary points on UV-irradiated samples with time variation. (e) shows the changes of D and G-peak intensity ratios as a function of UV-irradiation time.

Figure S6 (a-d) show the Raman spectra for six arbitrary points on 30 s, 45 s, 60 s, and 70 s UV irradiated graphene sheets, respectively. The Raman spectra for 30s UV-irradiated time differs significantly from one point to other. Meanwhile, in the 45 s UV-irradiation condition, we obtained very stable spectra distributions in a large area of the sample. However, further UV-irradiation exhibits decrease in intensity ratio of D and G-peaks, as shown in Fig S6 (e). Eckmann *et al*. introduced the identification of GO optimized condition using I_D_/I_G_ ratio analysis, [S2] and our Raman analysis of I_D_/I_G_ ratios as a function of UV irradiation time exhibits same tendency. As shown in Fig. S6e, we determine that the optimized condition of UV irradiation time is 45 s for uniform graphene oxidation.

**SUPPLEMENTARY REFERENCES**

S1. C. J Shearer, A. D. Slattery, A. J. Stapleton, J. G. Shapter, C. T. Gibson. Nanotechnology 27 125704 (2016)

S2. A. Eckmann, A. Felten, A. Mishchenko, L. Britnell, R. Krupke, K. S. Novoselov, and C. Casiraghi. Nano Lett, 12, 3925 (2012).
